# Phenol-soluble modulin α4 mediates *Staphylococcus aureus*-associated vascular leakage by stimulating heparin-binding protein release from neutrophils

**DOI:** 10.1038/srep29373

**Published:** 2016-07-07

**Authors:** Lin li, Yaya Pian, Shaolong Chen, Huaijie Hao, Yuling Zheng, Li Zhu, Bin Xu, Keke Liu, Min Li, Hua Jiang, Yongqiang Jiang

**Affiliations:** 1 State Key Laboratory of Pathogen and Biosecurity, Institute of Microbiology and Epidemiology, Academy of Military Medical Sciences, Beijing, China; 2 Key Laboratory of infection and immunity, Institute of Biophysics, Chinese Academy of Science, Beijing 100101, China; 3 Institution of Microbiology, Chinese Academy of Sciences, Beijing, China; 4 State Key Laboratory of Pathogen and Biosecurity, Beijing Institute of Biotechnology, Beijing 100071, China; 5 National Center of Biomedical Analysis, Beijing, China; 6 Department of laboratory medicine, Renji Hospital, School of Medicine, Shanghai Jiao Tong University, Shanghai, China

## Abstract

Vascular leakage frequently occurs in patients with severe *Staphylococcus aureus* infection. However, the mechanism underlying *S. aureus* infection-induced vascular leakage remains unclear. Here, we identified the *S. aureus* virulence factor phenol-soluble modulin (PSM)α4 from the culture supernatant of strain USA300 as a stimulator of heparin-binding protein (HBP) release from polymorphonuclear neutrophils (PMNs) and demonstrated that PSMα4-induced HBP release from PMNs leads to vascular leakage. PSMα4 appeared less cytolytic than PSMα1–3 and was insensitive to lipoproteins; it significantly increased myeloperoxidase and elastase release from PMNs and cell surface CD63 expression in PMNs. PSMα4-induced HBP release required formyl peptide receptor 2 (FPR2) and phosphoinositide 3-kinase (PI3K) and depended on Ca^2+^ influx and cytoskeleton rearrangement. Thus, PSMα4 may stimulate HBP release by activating FPR2 and PI3K to initiate PMN degranulation. PSMα4-induced HBP release from PMNs increased endothelial cell monolayer permeability *in vitro* and induced vascular leakage in mice. This novel function of PSMα4 may contribute to the pathogenesis of *S. aureus* and may be a potential therapeutic target.

Community-acquired methicillin-resistant *Staphylococcus aureus* (CA-MRSA) poses a serious threat to human health. CA-MRSA-associated infection can be local, such as skin and soft tissue infection, but sometimes leads to serious systemic infection, such as sepsis, necrotizing pneumonia, and toxic shock syndrome. The strong pathogenicity of CA-MRSA is associated with multiple virulence factors, including enterotoxins, hemolysins, bi-component leukocidins, and cytolytic peptides^1^. These virulence factors can contribute to vascular leakage and consequently lead to severe circulatory shock and multiple organ failure in patients with severe CA-MRSA infection. The molecular and cellular mechanisms underlying virulence factor-induced vascular leakage vary for different virulence factors. Staphylococcal enterotoxins (SEs) induce vascular leakage by stimulating T cells, and the massive production of cytokines by the stimulated T cells triggers a cytokine storm^2^,^3^. The virulence factors lipoteichoic acid and peptidoglycan induce tumor necrosis factor (TNF)-α secretion^4^, which has been shown to contribute to vascular leakage in a rat model of endotoxin-induced uveitis^5^. The binding of hemolysin α (Hla) to its receptor ADAM10 enhances the metalloprotease activity of ADAM10 to cleave endothelial cadherin, thus damaging the endothelial barrier function^6^,^7^. Panton–Valentine leukocidin lyses neutrophils to release cytotoxic granules and reactive oxygen metabolites, leading to pulmonary vascular damage^8^,^9^,^10^. Cysteine proteinases stimulate bradykinin release to induce vascular leakage^11^.

In addition to these well-recognized virulence factors, phenol-soluble modulin (PSM)α, which was originally identified from *Staphylococcus epidermidis*^12^, also is an important virulence factor of *S. aureus*. PSMα has multiple functions, including recruiting and lysing neutrophils^13^,^14^, lysing erythrocytes, and promoting biofilm formation^15^,^16^. Previous reports have shown that PSMα can exert its biological function only in serum-free conditions^16^. A clinical study showed that high level of serum PSMα antibody was associated with low risk of sepsis in patients with invasive *S. aureus* infection^17^. The mechanism underlying PSMα-associated pathogenesis remains unclear and it is unknown whether PSMα can induce vascular leakage.

Heparin-binding protein (HBP) is an early diagnostic marker for severe sepsis or septic shock caused by invasive bacterial infection^18^,^19^,^20^,^21^. It mediates neutrophil-evoked vascular leakage by inducing Ca^2+^-dependent cytoskeleton rearrangement in endothelial cells and promoting intercellular gap formation between endothelial cells^22^. HBP is stored in secretory vesicles and primary granules of human polymorphonuclear neutrophils (PMNs)^23^,^24^, and its release is stimulated by multiple mediators, including lipid leukotriene B4 (LTB4)^25^, M protein^26^,^27^, and streptolysin O (SLO)^28^, and results in vascular leakage.

We hypothesized that the *S. aureus* virulence factor PSMα may induce HBP release from PMNs, consequently inducing vascular leakage. This study aimed to test this hypothesis and to investigate the molecular mechanism underlying the effects of PSMα on PMNs.

## Results

### PSMα4 in the culture supernatant of *S. aureus* stimulates HBP release from whole blood of healthy donors

**Figure 1:**
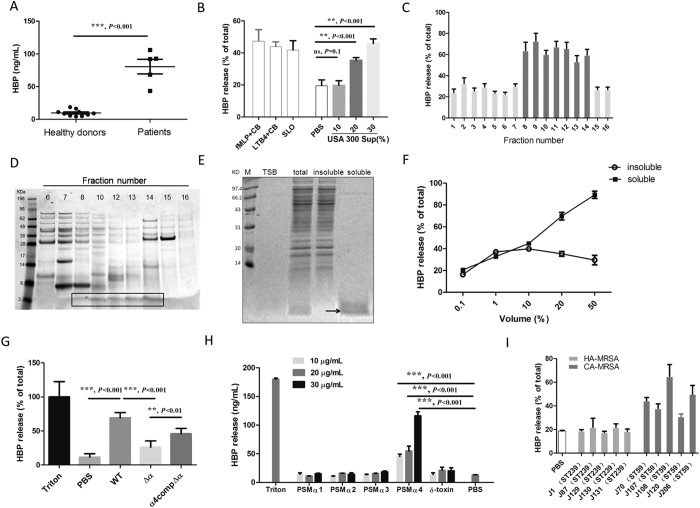
PSMα4 from *S. aureus* culture supernatant stimulates HBP release from whole blood of healthy donors. (**A**) Blood specimens from patients infected with *S. aureus* contained significantly higher levels of HBP than blood from healthy donors. (**B**) USA300 supernatant induced HBP release from whole blood in a dose-dependent manner (10%, 20%, and 30%). fMLP (100 nM) + cytochalasin B (5 μg/mL), LTB4 (20 ng/mL) + cytochalasin B (5 μg/mL), and SLO (10 μg/mL) were used as positive controls for HBP release. PBS was used as the negative control. (**C**) Fractions 8 to 14 eluted from a Resource Q column induced HBP release. (**D**) High-resolution SDS-PAGE revealed a unique peptide component (3 kDa) in the eluted fractions stimulating HBP release. (**E**) The small peptide was soluble in ethanol. (**F**) Ethanol-soluble peptides stimulated HBP release from whole blood in a dose-dependent manner, while ethanol-insoluble peptides weakly induced HBP release. (**G**) Supernatant from the PSMα deletion mutant (Δα) failed to induce HBP release. Supernatant from the complemented Δα strain (α4compΔα) showed normal HBP release. (**H**) Synthetic PSMα4 peptide stimulated HBP release in a dose-dependent manner while PSMα1–3 did not stimulate HBP release from whole blood. (**I**) Culture supernatant of CA-MRSA stimulated HBP release to a higher extent than that of HA-MRSA. CA-MRSA strain numbers: 1, 87, 129, 130, and 131. HA-MRSA strain numbers: 70, 107, 108, 120, and 206. ***P < 0.001, **P < 0.01, *P < 0.05.

HBP levels were significantly higher in the blood specimens from patients severely infected by *S. aureus* than in those of healthy donors (*P* < 0.001, Fig. 1A). *In vitro* assay showed that the culture supernatant of *S. aureus* directly induced HBP release from the whole blood of healthy donors in a dose-dependent manner (Fig. 1B). Consistent with previous reports, formyl-methionyl-leucyl-phenylalanine (fMLP), SLO, and LTB4 stimulated HBP release from whole blood (Fig. 1B).

Several well-known *S. aureus* virulence factors, including the superantigens SEA and SEB, toxic shock syndrome toxin (TSST)-1, Hla, lipoteichoic acid and PVL did not induce HBP release from whole blood of healthy donors (Supplementary Fig. 1). Proteinase K treatment of the USA300 culture supernatant abolished HBP release (Supplementary Fig. 2A). However, heat treatment of the culture supernatant did not significantly affect its ability to induce HBP release (Supplementary Fig. 2B), indicating that the molecules mediating HBP release may be heat-resistant proteins or peptides. The crude bacterial culture supernatant extract was fractionated on a Resource Q column; the middle- and late-eluting fractions (8 to 14) induced HBP release (Fig. 1C). High-resolution sodium dodecyl sulfate-polyacrylamide gel electrophoresis (SDS-PAGE) analysis revealed that the unique peptide component in the fractions that stimulated HBP release was approximately 3 kDa (Fig. 1D). Next, we extracted the peptides ≤10 kDa from the supernatant. The ethanol-soluble peptides were approximately 3 kDa (Fig. 1E) and induced HBP release in a dose-dependent manner (Fig. 1F). The ethanol-insoluble peptides (≥10 kDa) only slightly induced HBP release (Fig. 1F).

Using Electrospray Ionization Mass Spectrometry (ESI-MS), 6 proteins, including 3 PSMα proteins, were identified from the ethanol-soluble peptides (Table 1). PSMα has been recently proposed as an important virulence factor of *S. aureus*^14^. The culture supernatant of the PSMα deletion mutant Δα failed to induce HBP release, whereas complementation of the Δα strain with PSMα4 α4compΔα restored HBP release significantly (*P* < 0.05, Fig. 1G). To confirm the role of PSMα4 in the stimulation of HBP release, PSMα1–4 peptides were synthesized. Only PSMα4 stimulated HBP release from whole blood in a dose-dependent manner (all *P* < 0.05, Fig. 1H).

The capacity to induce HBP release varied among different *S. aureus* strains. Previous report has shown that CA-MRSA strains are more virulent than HA-MRSA strains because of higher expression of PSMα in CA-MRSA than in HA-MRSA strains^14^. Here both PSMα mRNA and protein expression levels were dramatically higher in the five CA-MRSA strains than HA-MRSA strains(Supplementary Fig. 3). Accordingly, the culture supernatant of CA-MRSA also stimulated HBP release to a higher extent than that of HA-MRSA (Fig. 1I). Thus, the CA-MRSA strain USA300^29^ was used in the rest of this study.

### PSMα4 stimulates primary granule exocytosis, which is free of the blocking effect of serum lipoprotein

**Figure 2:**
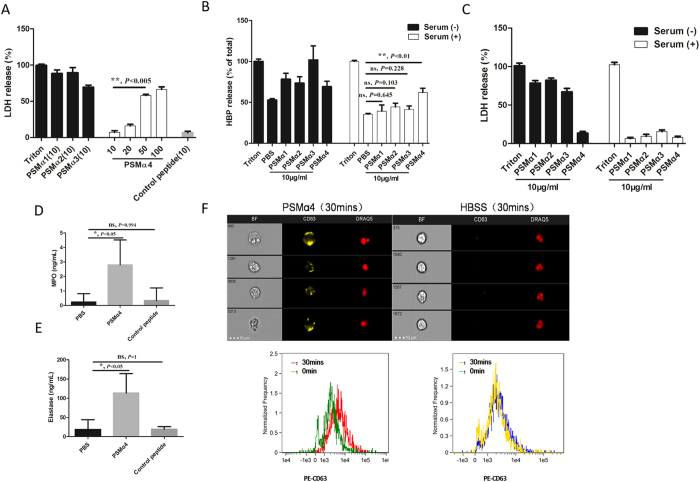
PSMα4 stimulates primary granule exocytosis, which is free of the blocking effect of serum lipoprotein. (**A**) Synthetic PSMα4 appeared to be less cytotoxic than PSMα1–3. Cytotoxicity of PSMα1–4 on PMNs was evaluated LDH test. PMNs were incubated with PSMα1–3 at 10 μg/mL, PSMα4 at 10, 20, 50, or 100 μg/mL at 37 °C for 30 min. The control peptide was added at 10 μg/mL. (**B**) Human serum (10%) significantly reduced PSMα1–3-induced HBP release from PMNs but did not affect PSMα4-induced HBP release from PMNs. (**C**) Serum significantly reduced PSMα1–3-induced PMN lysis but had no effects on PSMα4-induced PMN lysis. (**D**) PSMα4 peptide (10 μg/mL) increased MPO release from whole blood. (**E**) PSMα4 peptide (10 μg/mL) increased elastase release from whole blood. MPO and elastase in the supernatant were analyzed by ELISA. (**F**) PSMα4-induced cell surface expression of CD63 was not affected by human serum (10%). PMNs were incubated with PSMα4 peptides (10 μg/mL) with 10% human serum at 37 °C for 30 min. The PMNs were stained with PE-labeled anti-CD63 antibody (1:100 dilution) and 5 μM DRAQ5. ***P < 0.001, **P < 0.01, *P < 0.05.

PSMα1–3 can dramatically induce PMN lysis, and synthetic PSMα4 exerts much lower cytolytic effect on PMNs than PSMα1–3. Synthetic PSMα4 at ≥50 μg/mL exerted a substantial cytolytic effect on PMNs (Fig. 2A) (P < 0.05, Fig. 2A). Serum high-density lipoprotein (HDL) can block the cytolytic effects of PSMα1–3 on PMNs^16^. Thus, PSMα1–3 cannot effectively exert their biological functions *in vivo*. In this study, 10% human serum significantly reduced PSMα1–3-induced HBP release from PMNs (*P* > 0.05, Fig. 2B), and the HBP release was most likely associated with PMN lysis (*P* > 0.05, Fig. 2C). Human serum (10%) had no blocking effect on PSMα4-induced HBP release (*P* < 0.05, Fig. 2B). Further study showed that PSMα4 at 10 μg/mL also significantly stimulated the release of myeloperoxidase (MPO) (Fig. 2D) and elastase (Fig. 2E) in whole blood and increased the expression of cell surface CD63 in PMNs in the presence of serum (Fig. 2F). In addition, confocal microscopy demonstrated that PSMα4 induced the translocation of CD63 from the cytoplasm to the cell surface (Supplementary Fig. 4). Because MPO and elastase are primary granule proteins, upregulation of CD63 on the cell surface and increased levels of MPO and elastase in the extracellular environment indicated that PSMα4 induces PMN degranulation. The fact that only PSMα4 caused PMN exocytosis in the presence of serum lipoprotein suggests that this molecule has a unique function during infection *in vivo*.

### PSMα4 stimulates HBP release from PMNs via formyl peptide receptor 2 (FPR2) and activation of PI3K signaling pathway

**Figure 3:**
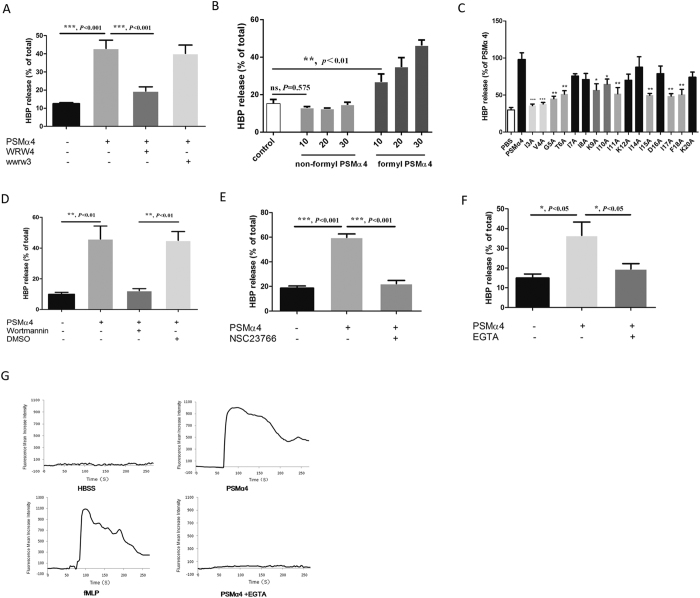
PSMα4 stimulates HBP release from PMNs via FPR2 and PI3K signaling pathway. (**A**) FPR2 antagonist WRW4 significantly reduced HBP release from whole blood. Whole blood was pre-incubated with WRW4 (50 μg/mL) or the scramble all d-amino acid control wwrw3 (50 μg/mL) at 37 °C for 15 min^30^ and then treated with PSMα4 (10 μg/mL) at 37 °C for 30 min. HBP in the supernatant was analyzed by ELISA. (**B**) Formyl PSMα4 peptide induced HBP release dose-dependently. Human whole blood was incubated with 10, 20, or 30 μg/mL non-formyl or formyl PSMα4 at 37 °C for 30 min. PBS was used as the negative control. (**C**) The critical amino acid residues of PSMα4 to induce HBP release were screened by alanine substitution. The concentration of the peptides was 10 μg/mL. Replacement of I3, V4, G5, T6, I11, I15, I17, or F18 with alanine significantly abolished HBP release. (**D**) PI3K-specific inhibitor wortmannin completely abolished HBP release. (**E**) Rac-specific inhibitor NSC23766 completely abolished HBP release. Whole blood was pre-incubated with 1 μM wortmannin or 50 μM NSC23766 at 37 °C for 1 h and then treated with PSMα4 (10 μg/mL) for 30 min. HBP in the supernatant was analyzed by ELISA. (**F**) EGTA in culture media significantly reduced HBP release. Human whole blood was pre-treated with 20 mM EGTA at 37 °C for 15 min and then stimulated with PSMα4 (10 μg/mL). (**G**) PSMα4 induced Ca^2+^ influx into PMNs. Fluo-3/AM was loaded to PMNs before stimulation with PSMα4. fMLP (1 μM) was used as a positive control for Ca^2+^ influx.

FPR2 has been found to play a critical role in PSMα-induced PMN activation and chemotaxis^30^,^31^. In the current study, the FPR2 antagonist WRW4 significantly abolished PSMα4-induced HBP release from PMNs, whereas the control peptide, wwrw3, had no effect (Fig. 3A). In addition, synthetic non-formyl PSMα4 failed to induce HBP release, whereas formyl PSMα4 induced HBP release in a dose-dependent manner (*P* < 0.05, Fig. 3B). Thus, PSMα4 amino (N) terminus formylation is required for induction of HBP release. The amino acid residues in PSMα4 that might be critical to HBP release induction were screened using alanine substitution^32^. Replacement of I3, V4, G5, T6, I11, I15, I17, and F18 with alanine significantly abolished HBP release (Fig. 3C).

To investigate the molecular mechanism underlying PSMα4-induced HBP release further, FPR2 downstream signaling molecules including phospholipase C (PLC), phosphoinositide 3-kinase (PI3K) and Src kinase were examined. The PLC-specific inhibitor U-73122 and the Src-specific inhibitor PP2 had no effect on PSMα4-induced HBP release (data not shown); however, the PI3K-specific inhibitor wortmannin significantly reduced HBP release (*P* < 0.05, Fig. 3D). These results suggested that PSMα4 may activate the PI3K signaling pathway to induce PMN degranulation. Degranulation is often associated with cytoskeletal rearrangement and Rac has been recognized to regulate cytoskeletal rearrangement. The Rac-specific inhibitor NSC23766 completely abolished PSMα4-induced HBP release (Fig. 3E). Wortmannin and NSC23766 had no cytotoxic effects on PMNs (Supplementary Fig. 5). In addition to cytoskeletal rearrangement, Ca^2+^ influx is required for PSMα4-induced HBP release. The Ca^2+^ chelator EGTA significantly reduced HBP release (Fig. 3F). PSMα4 stimulated Ca^2+^ influx in PMNs, and EGTA in culture media completely blocked the PSMα4-induced Ca^2+^ influx (Fig. 3G).

### PSMα4-induced HBP release increases human umbilical vein endothelial cell (HUVEC) monolayer permeability

**Figure 4:**
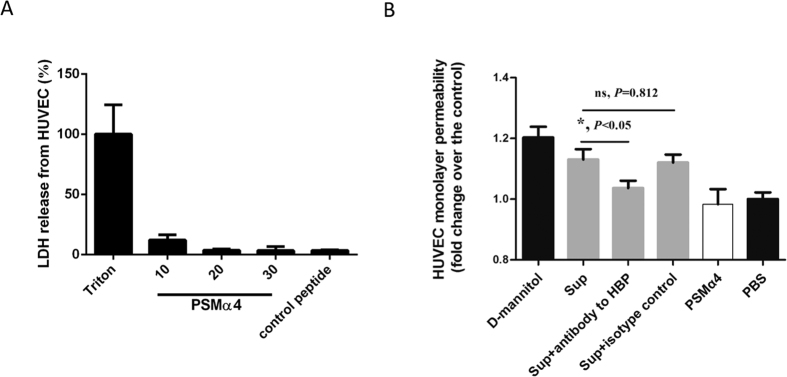
PSMα4-induced HBP release increases HVUEC monolayer permeability. (**A**) PSMα4 did not cause cytotoxicity in HUVECs. HUVECs (10^4^/well) were seeded in 24-well plates and incubated for 24 h. The HUVECs were then incubated with PSMα4 at 10, 20, or 30 μg/mL at 37 °C for 30 min. The supernatant was collected and cytotoxicity was analyzed by LDH assay. (**B**) PSMα4-induced HBP release increased HVUEC monolayer permeability. The HUVEC monolayer on the transwell insert chamber was incubated with d-mannitol (1.4 mM), culture supernatant of whole blood treated with PSMα4 (10 μg/mL), the supernatant + functional blocking antibody against HBP, the supernatant + the isotype control IgG (50 μg/mL), PSMα4 (10 μg/mL), or PBS at 37 °C for 30 min. Lucifer yellow was also added to the incubation media. The fluorescence intensity in the lower chamber of the transwells was determined. d-mannitol and PBS were used as the positive and negative control, respectively.

HBP is a potent inducer of vascular leakage. Thus, PSMα4-induced HBP release from PMNs might result in vascular leakage. Synthetic PSMα4 peptide did not show cytotoxic effects on HUEVCs (Fig. 4A). HUVEC monolayer permeability was not directly affected by synthetic PSMα4 peptide alone (Fig. 4B). However, the culture supernatant of whole blood treated with PSMα4 peptide significantly increased the HUVEC monolayer permeability, while functional blocking antibody against HBP significantly blocked the increased permeability (*P* < 0.05, Fig. 4B). These results suggested that PSMα4-induced HBP release might increase the permeability of the HUVEC monolayer. A previous study has shown that PSMα induces TNF-α and IL-8 release from PMNs^14^, and both TNF-α and IL-8 affect endothelial barrier dysfunction^33^. Here, PSMα-4 at 10 μg/mL did not increase the release of TNF-α, IL-8, and IFN-γ from whole blood (data not shown). Thus, TNF-α, IL-8, and IFN-γ may not contribute to the increased HUVEC monolayer permeability stimulated by the PSMα4 peptide-treated whole-blood supernatant. PSMα4 also stimulated reactive oxygen species (ROS) production from PMNs, and 10% human serum only partially inhibited this (Supplementary Fig. 6). However, the permeability-enhancing effect of the ROS was not as large as that of HBP, indicating that the ROS did not play a critical role.

### PSMα4 induces vascular leakage in mice

**Figure 5:**
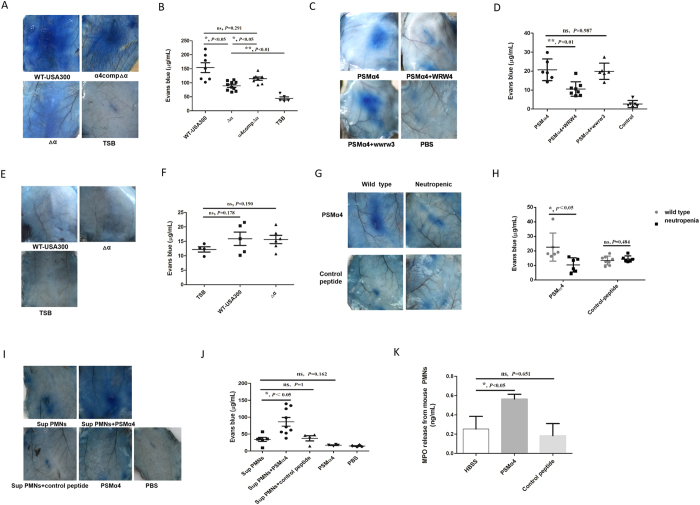
PSMα4 induces vascular leakage *in vivo.* (**A,B**) The supernatants of the wild-type USA300 and α4compΔα strains induced vascular leakage to a significantly higher extent than the supernatant of the Δα strain. Supernatant (0.1 mL) was injected intradermally into mice. TSB (0.1 mL) was injected as a negative control. (**C,D**) Synthetic PSMα4 induced vascular leakage via FPR2. PSMα4 peptide induced vascular leakage in mice via FPR2. The FPR2 antagonist WRW4 and the control wwrw3 were intravenously injected into mice (7.5 mg/kg) 30 min before intradermal injection of 100 μL PSMα4 peptide (10 μg/mL). PBS was injected as a negative control. The mice were intravenously injected with 100 μL Evans blue dye solution (1–2.5% in PBS) 3 h after intradermal injection of stimuli. The mice were scarified 30 min after Evans blue injection. Evans blue in mouse tissue was extracted and quantified. (**E,F**) The supernatant of the wild-type USA300 and Δα strains did not induce vascular leakage in neutropenic mice. Cyclophosphamide (1250 mg/kg) was administered intraperitoneally to mice to induce neutropenia 72 h before intradermal injection of the supernatant. The PMN count in neutropenic mice was <200 × 10^6^/L. Supernatant (0.1 mL) was injected intradermally into mice. TSB (0.1 mL) was injected as a negative control (**G,H**) Synthetic PSMα4 did not induce vascular leakage in neutropenic mice. Neutropenic mice were injected intradermally with 100 μL of PSMα4 peptide (10 μg/mL) and control peptide (KAFIDIIAKIIKIITGVIAM) (10 μg/mL). (**I,J**) The supernatant of mouse PMNs stimulated by PSMα4 induced vascular leakage in neutropenic mice. Mouse PMNs (purity >90%, 10^6^/mL) were incubated with HBSS, PSMα4 (10 μg/mL) or control peptide (KAFIDIIAKIIKIITGVIAM) (10 μg/mL) at 37 °C for 30 min, and then 0.1 mL of the respective supernatants was injected into neutropenic mice intradermally. (**K**) Synthetic PSMα4 peptide stimulated MPO release from mouse PMNs. Mouse PMNs (10^6^/mL) were incubated with PSMα4 (10 μg/mL) at 37 °C for 30 min. MPO release was evaluated by ELISA.

The Miles assay is frequently used to evaluate vascular leakage *in vivo*^11^,^22^. The culture supernatant of the PSMα deletion mutant Δα induced significantly less vascular leakage than that of the wild-type USA300 in female C57BL/6 mice, whereas the supernatant of α4compΔα induced vascular leakage to a significantly higher extent than that of Δα (Fig. 5A,B). These results indicated that PSMα4 might mediate vascular leakage in mice. Indeed, synthetic PSMα4 significantly increased vascular leakage, while the FPR2 antagonist WRW4 significantly reduced PSMα4-induced vascular leakage in mice (Fig. 5C,D).

Mouse neutrophils have been found to release a functional homolog of HBP^26^. Thus, we reasoned that depletion of mouse neutrophils would abolish PSMα4-induced release of the HBP functional homolog and the consequent vascular leakage. To test this hypothesis, we depleted mouse neutrophils by intraperitoneal injection of cyclophosphamide^34^,^35^. In the neutropenic mice, neither the supernatant of the wild-type strain nor that of strain Δα induced vascular leakage (Fig. 5E,F). Similarly, synthetic PSMα4 failed to induce vascular leakage in neutropenic mice (Fig. 5G,H). To confirm that PSMα4-induced vascular leakage was mediated by factors secreted from PMNs, we injected culture supernatant of mouse PMNs into the neutropenic mice. The supernatant of PSMα4-stimulated mouse PMNs significantly increased vascular leakage when compared with the controls, whereas the supernatant of mouse PMNs that were incubated with the control peptide did not (Fig. 5I,J). These results indicated that factors secreted from PSMα4-stimulated mouse PMNs might induce vascular leakage. Because PSMα4 did not induce TNF-α and IL-8 release from mouse whole blood (data not shown), the two cytokines may not directly contribute to the PSMα4-induced vascular leakage in mice. PSMα4 significantly increased MPO release from mouse PMNs (Fig. 5K), indicating that PSMα4 might stimulate degranulation in mouse PMNs.

### Wild-type *S. aureus* strain induces a greater level of plasma leakage in the lung tissue than the Δα strain

**Figure 6:**
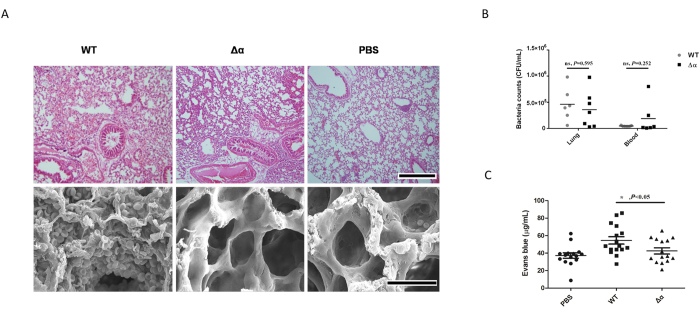
Wild-type *S. aureus* strain induces a greater level of plasma leakage in lung tissue than the Δα strain. (**A**) Mice infected with wild-type strain (1 × 10^7^ CFU/mouse) showed a greater amount of erythrocytes in the lungs than mice infected with Δα (1 × 10^7^ CFU/mouse). Microscopic images (200×) of H&E-stained lung tissue sections and electron microscopic images of lung tissue sections from mice infected with the wild-type or Δα are presented. PBS was used as the negative control. The scale bar represents 500 μm for light microscopy and 30 μm for scanning electron microscopy. Evans blue effusion in the lung tissue was significantly higher in mice infected with the wild-type strain than in mice infected with the Δα strain. (**B**) The bacterial count in the blood and lungs was not significantly different between the 2 groups of mice. The bacteria in blood and lungs were counted by gradient dilution and the flat colony counting method. (**C**) Evans blue effusion in the lung tissue was significantly higher in mice infected with wild-type strain than in mice infected with the Δα strain. ***P < 0.001, **P < 0.01, *P < 0.05.

When mice were infected intravenously with 10^7^ USA300 or USA300Δα, no mortality occurred in the two groups (data not shown). However, compared with lung tissue of mice infected with the Δα strain, the lung tissue of mice infected with the wild-type strain showed more severe plasma leakage, which was characterized by erythrocyte and plasma effusion from the capillaries into the lung tissue (Fig. 6A). The bacterial counts in the blood and lungs were not significantly different between the 2 groups of mice (Fig. 6B). Evans blue effusion in the lung tissue was significantly higher in mice infected with wild-type strain than in mice infected with the Δα strain (*P* < 0.05, Fig. 6C), indicating more severe plasma leakage in mice infected with the wild-type strain.

## Discussion

The current study revealed that PSMα4 secreted by *S. aureus* stimulated HBP release from PMNs and consequently caused vascular leakage *in vivo* in a mouse model. Previous studies have shown that PSMα1–3 have strong cytolytic ability and lyse PMNs efficiently at low concentration^13^,^14^. Particularly, *S. aureus* PSMα3 is the most potent cytolytic so far^13^,^14^. However, we found that PSMα4 elicited release of HBP from PMNs by granule exocytosis rather than cytolysis.

We observed that serum did not block PSMα4-induced HBP release from PMNs, while it significantly reduced PSMα1–3-induced PMN lysis and HBP release from PMNs. The report by Surewaard *et al*.^16^ showed that lipoproteins that are commonly found in human serum, such as high, low, and very low-density lipoproteins, inhibit PSMα3-induced PMN lysis in a dose-dependent manner. Thus, although PSMα4 appears to be less cytolytic than PSMα1–3, it may alter the host response to a greater extent than PSMα1–3 in a serum enviroment because of its resistance to lipoprotein-mediated neutralization. Indeed, we found that PSMα4 but not PSMα1–3 induced HBP release from cells in whole blood in a dose-dependent manner. Alanine-replacement screening revealed that some amino acid residues in PSMα4 were critical for stimulation of HBP release. These critical residues may be associated with the resistance of PSMα4 to lipoproteins or its receptor-binding ability. The critical residues may also be potential therapeutic targets.

PSMα4 induced HBP release from PMNs possibly by degranulation as the levels of protein markers for primary granules including MPO, elastase, and cell surface CD63 were significantly increased by PSMα4. Although it shows some similar signal moleculars with fMLP induced activation of PMN, one of the difference is that PSMα4 stimulate HBP release without priming neutrophil with cytochalasin B which is needed by fMLP. That means, PSMα4 may cause more intense or different signal pathway through binding FPR2. Previous studies have demonstrated some similar mechanisms of HBP release from PMNs. LTB4 activated BLT1 receptors to induce HBP release from PMNs by PI3K-dependent degranulation^25^. In the current study, LTB4 release from whole blood was not stimulated by PSMα4 (data not shown). Thus, LTB4 may not be involved in PSMα4-induced HBP release from PMNs. M protein from *Streptococcus pyogenes* binds integrin β2 to stimulate HBP release from neutrophils by degranulation^26^,^27^. *S. pyogenes* SLO perforates human PMNs and induces Ca^2+^ influx and p38 MAPK activation, consequently inducing HBP release^28^. So the signal pathway involved in HBP release is complicated. More details of signal regarding PSMα4-induced HBP release needs further study.

Vascular integrity is crucial for normal physiological function of the human body. HBP plays a critical role in mediating neutrophil-induced vascular leakage; thus, it is considered a potent inducer of vascular leakage^22^. We found both *in vitro* and *in vivo* evidence supporting that PSMα4 alone cannot induce vascular leakage. Synthetic PSMα4 peptide alone failed to increase HUVEC monolayer permeability, and injection of wild-type USA300 supernatant did not increase Evans blue leakage in neutropenic mice. On the other hand, functional blocking antibody against HBP significantly reduced the PSMα4-stimulated HUVEC monolayer leakage, and injection of supernatant of mouse PMNs stimulated with PSMα4 increased vascular leakage in neutropenic mice. These results support that PSMα4-induced HBP release from PMNs actually results in vascular leakage. The role of PSMα4 in the stimulation of vascular leakage was confirmed by the results of the mouse infection model; red blood cells were clearly present in the lungs of mice infected with the wild-type strain, whereas they were absent in the lungs of mice infected with the PSMα deletion strain. Though HBP was the most potential vascular leakage inducer, there were a lot of proteolytic and redox active enzymes known to directly or indirectly impair endothelial cell junctional integrity (thus increasing vascular permeability) being released by activated PMNs. The relationship of PSMα4 and these factors would be studied further.

In summary, PSMα4 was demonstrated to play a critical role in response to neutrophils in blood during infection, which implys PSMα4 may contribute to the research of *S. aureus* pathogenesis and need more attention.

## Methods

### Human experiment ethical statement

For experiments involving human blood samples, signed informed consent was obtained from all the patients or their guardians and healthy volunteers. All the experimental methods were carried out in accordance with the approved guidelines of Institutional Medical Ethics Committee of AMMS (Academy of Military Medical Sciences). All the protocols for handling patients’ or healthy donors’ blood specimens were approved by the Institutional Medical Ethics Committee of AMMS.

### Animal experiment ethical statement

All experimental procedures involving mice were carried out in strict accordance with the recommendations in the *Guide for the Care and Use of Laboratory Animals* of the National Institutes of Health and State Key Laboratory of Pathogens and Biosecurity of the Institute of Microbiology and Epidemiology. The protocol for animal handling and experiment was approved by the Institutional Review Board of Academy of Military Medical Science (approval number: IACUC of AMMS-2014-032).

### Antibodies and reagents

Recombinant HBP (Catalog Number: 2200-SE-050/CF), polyclonal goat anti-human HBP antibody (Catalog Number: AF2200) and monoclonal mouse anti-human HBP antibody (Catalog Number: MAB2200) were purchased from R&D Systems (Minneapolis, MN, USA). EGTA(Catalog Number: E3889), U73122(Catalog Number: U6756), PP2(Catalog Number: P 0042), wortmannin (Catalog Number: F9128) were purchased from Sigma-Aldrich (St. Louis, MO, USA). NSC23766 (Catalog Number: sc-204823) was purchased from Santa Cruz Biotechnology.

### Blood specimens from patients

Blood specimens from 5 patients who were infected with *S. aureus* and presented with septic shock, acute cellulitis, and respiratory failure were kindly provided by the Chinese Center for Disease Control and Prevention. The specimens were anonymized. Additionally, blood was collected from 10 healthy donors in the 307^th^ Hospital of Chinese People’s Liberation Army.

### Bacteria and plasmids

*S. aureus* strain USA300^29^ and its isogenic ΔPSMα deletion mutant (Δα) were gifts from Dr. Ming Li (Shanghai Jiao Tong University, Shanghai, China). A complementary plasmid for PSMα4, PRB473_*PSMα4*_, was constructed as described previously^36^,^37^. The primer sequences (including restriction enzyme sites) for the *PSMα4* promoter and gene for constructing PRB473_*PSMα4*_are listed in Table 2. The *PSMα4* promoter and gene fragments were fused using overlapping PCR. The complementary plasmid was transformed into *S. aureus* RN4220 and then the Δα strain. The bacteria containing PRB473_*PSMα4*_(α4compΔα strain) were screened on tryptic soy agar (Becton Dickinson, Franklin, NJ, USA) plates supplemented with chloromycetin (20 μg/mL). Δα strain containing the empty plasmid PRB473 was used as the control. The expression of PSMα4 in the α4compΔα strain was confirmed by RT-PCR and ESI-MS. The *S. aureus* strains HA-MRSA (J1, J87, J129, J130, and J131) and CA-MRSA (J70, J107, J108, J120, and J206) were kindly provided by Dr. Li Han (Institute of Disease Control and Prevention, Academy of Military Medical Science, Beijing, China). Both strains had been isolated from Chinese patients.

### Synthetic peptides

The peptides PSMα1–4 were synthesized according to the previous report^14^ by Sangon Biotech (Shanghai, China). The purity of the peptides was >95%. PSMα4 were synthesized in its formylated and non-formylated form. The control peptide sequence is KAFIDIIAKIIKIITGVIAM^30^. The FPR2/ALX inhibitor WRW4 (WRWWWW) and its scrambled variant wwrw3 with all-d amino acids (wwrwww)^30^ were synthesized by Sangon Biotech. fMLP with a purity ≥95% was purchased from Sigma-Aldrich.

### Protein and peptide purification from *S. aureus* supernatant

Strain USA300 was aerobically cultured overnight in tryptic soy broth (TSB) at 37 °C. The bacterial culture was centrifuged at 5000 × *g* for 30 min and the supernatant was collected. Ammonium sulfate was added to the supernatant to achieve 75% saturation. The mixture was incubated at 4 °C for 4 h, and the precipitate was collected after centrifugation at 5000 g for 30 min, dissolved in buffer A (20 mM Tris-HCl, pH 8.0), and dialyzed against buffer A. The crude extract was loaded on a Resource Q column (GE Healthcare Life Sciences, Pittsburgh, PA, USA). The column was eluted with buffer B containing a linear salt gradient (20 mM Tris-HCl, 0–1 M NaCl, pH 8.0). The eluted fractions were analyzed by SDS-PAGE. To isolate peptides, the bacterial culture supernatant was mixed with 100% ethanol at 4 °C to reach a final ethanol concentration of 80% (v/v), and the mixture was centrifuged at 1000 × *g* at 4 °C for 15 min^38^. The ethanol-soluble fraction was dried in an incubator at 50 °C. The purity of the ethanol-soluble and insoluble fractions was analyzed on pre-cast gel (Novex, Shanghai, China).

### Isolation of PMNs

Human PMNs were isolated from fresh blood (sodium citrate anticoagulant was added) of healthy volunteers as described previously^26^. The isolated PMNs were suspended in Hank’s balanced salt solution (HBSS) (with Ca^2+^ and Mg^2+^). Mouse PMNs were isolated from peripheral blood using the TBD kit (TBD sciences, Tianjin, China) following the manufacturer’s instruction. Briefly, whole blood was withdrawn from the orbital venous plexus of the mice and diluted with the dilution buffer from the kit in a 1:1 ratio, and then overlaid on the gradient buffer from the kit. After centrifugation at 680 × *g* for 30 min, the neutrophil layer was collected and red blood cells were removed using lysis buffer. Mouse PMNs were suspended in HBSS (with Ca^2+^ and Mg^2+^).

### Measurement of HBP release

Fresh whole blood (100 μL) or isolated PMNs (1 × 10^6^) were incubated with different stimuli in a final volume of 1.0 mL at 37 °C for 30 min. Then, the supernatants were collected by centrifugation. The HBP in the supernatant was measured by a double-antibody sandwich assay (antibodies AF2200 and MAB2200 from R&D Systems). To measure total HBP, 100 μL of whole blood was diluted in PBS and treated with 0.5% Triton X-100.

### Measurement of secreted MPO, elastase, TNF-α, IL-8, IFN-γ and LTB4

Fresh whole blood or isolated PMNs were incubated with the different stimuli at 37 °C for 30 min, and culture supernatants were collected by centrifugation. MPO, elastase, TNF-α, IL-8, IFN-γ, and LTB4 in the culture supernatants were analyzed by ELISA. The ELISA kits for human MPO, human elastase, and mouse MPO were from Abcam (USA). The ELISA kits for mouse TNF-α, mouse IL-8, human TNF-α, human IL-8, and human IFN-γ were purchased from NeoBioscience (Shenzhen, China). The ELISA kit for LTB4 was from Cayman Chemical Company (Ann Arbor, MI, USA).

### Flow cytometry and confocal microscopy to detect CD63

Flow cytometry to detect CD63 was conducted according to a previous report^28^. Briefly, PMNs (2 × 10^6^/mL) were stimulated with different stimuli at 37 °C for 30 min. The PMNs were collected by centrifugation and incubated with mouse anti-human CD63 antibody conjugated with PE (BD Pharmingen) and 5 μM DRAQ5 at 37 °C for 30 min. After washing, the PMNs were analyzed by imaging flow cytometry (Merck Millipore, USA). Neutrophil degranulation was analyzed by confocal microscopy according to a previous report^39^. Briefly, PMNs were collected, fixed in 4% (v/v) paraformaldehyde on ice for 40 min, and permeabilized with 0.2% Triton X-100 for 30 min. After washing, the PMNs were incubated with mouse monoclonal antibody against CD63 (BD Pharmingen) at room temperature for 1 h, washed with HBSS, and incubated with goat anti-mouse immunoglobulin G (IgG) conjugated to tetramethyl rhodamine-5-(and-6)-isothiocyanate. The labeled PMNs were observed under a confocal microscope (Olympus FV1000, Japan).

### PSMα4 cytotoxicity evaluation

PSMα4 cytotoxicity to neutrophils and HUVECs was evaluated by using a lactate dehydrogenase cytotoxicity detection kit (CytoTox 96 Non-Radioactive Cytotoxicity Assay; Promega, USA) according to the manufacturer’s protocol.

### Measurement of ROS production

ROS production was measured by using dihydrorhodamine 123 (Santa Cruz Biotechnology, Dallas CA, USA). PMNs (1 × 10^6^) were incubated with different stimuli at 37 °C for 30 min, collected by centrifugation at 400 × *g* for 10 min, and resuspended in HBSS. Dihydrorhodamine 123 was added to the PMN suspension at a final concentration of 1 μM, and the PMN suspension was incubated for 30 min and then washed twice. Then, the cells were collected and analyzed by flow cytometry (Accuri C6; Becton Dickinson).

### Intracellular calcium measurement

PMNs (2–5 × 10^6^/mL) were incubated with 1 μM fluo-3/AM in HBSS (with Ca^2+^ and Mg^2+^) in the dark at 37 °C for 30 min. Then, the PMNs were washed twice and resuspended in HBSS. The fluo-3/AM-loaded PMNs were incubated with or without EGTA for 30 min, and then with different stimuli at 37 °C for 30 min. The fluorescence emission of fluo-3/AM was measured using a confocal microscope (Olympus FV1000; Olympus, Japan).

### Endothelial permeability assay

Endothelial monolayer permeability was evaluated as previously described^40^. HUVECs were seeded in transwell inserts at a density of 5 × 10^4^/200 μL/insert. After the HUVECs reached 100% confluency, Lucifer yellow (400 μM), and different stimuli were added. d-mannitol was used as a positive control to measure endothelial permeability. After a 30-min incubation with the dye and stimuli or d-mannitol, the fluorescence intensity in the lower chamber of the transwells was determined by Varioskan Flash Multiplate Reader (Thermo Scientific USA).

### Vascular leakage assay

Female C57BL/6 mice (age, 8–10 weeks) were purchased from the Animal Center of the Academy of Military Medical Science. The mice were randomized into different treatment groups. One hundred microliters TSB or culture supernatant (12 h) of the wild-type, Δα, or α4compΔα bacterial strains was injected intradermally into mice. To investigate the role of FPR2 in PSMα4-induced vascular leakage, WRW4 was intravenously injected into mice (7.5 mg/kg) 30 min before injection of 100 μL of PSMα4 peptide (20 μg/mL)^30^. To examine the role of neutrophils in PSMα4-induced vascular leakage, neutropenia was induced in mice by intraperitoneal injection of 250 μL of cyclophosphamide (250 mg/mL) 72 h before intradermal injection of other stimuli. Mouse PMNs were stimulated with PSMα4 or the control peptide, and their culture supernatant was injected intradermally into the neutropenic mice. Three hours after injection of the stimuli, the mice were intravenously injected with 100 μL Evans blue dye solution (1–2.5% in PBS). The mice were scarified 30 min after Evans blue injection. Evans blue in mouse tissue was extracted and quantified^41^.

### Histopathological examination

Mice were injected intravenously with the wild-type or Δα bacterial strain at 1 × 10^7^ CFUs/mouse. PBS was injected as a negative control. Survival rate was recorded for 24 h. The mice were sacrificed 24 h after bacterial injection, and the lungs were immediately removed and fixed in formalin. The tissue samples were sectioned and stained with hematoxylin and eosin (H&E) dye. The tissue sections were examined by pathologists. Tissue specimen preparation for scanning electron microscopy (S-3400N, Hitachi, Japan) was performed based on a previous report^26^.

### Statistical analysis

Data are presented as the mean ± standard deviation. The statistical analysis software SPSS 19.0 was used to analyze the data. One-way ANOVA or Student’s *t*-test was performed to compare multiple groups or 2 groups, respectively. A 2-sided *P* < 0.05 was considered statistically significant.

## Additional Information

**How to cite this article**: li, L. *et al*. Phenol-soluble modulin α4 mediates *Staphylococcus aureus*-associated vascular leakage by stimulating heparin-binding protein release from neutrophils. *Sci. Rep.* **6**, 29373; doi: 10.1038/srep29373 (2016).

## Supplementary Material

Supplementary Information

## Figures and Tables

**Table 1:** Peptide identification from ESI-MS.

Protein name	Mass (Da)	Score	Total independent spectra	Cover length (%)
Hypothetical protein MW1056	4493	651	10	79
Cytosolic toxin PSM alpha1	2258	562	32	90
Cytosolic toxin PSM alpha2	2276	524	17	90
Hypothetical protein	9141	261	3	25
Delta-hemolysin	5063	208	8	57
Cytosolic toxin PSM alpha4	2170	180	4	90

**Table 2:** Primer sequences for PSMα4 and PSMα4 promoter.

Construction of PRB473_*PSMα4*_Sequence (5′-3′)	
Promoter sequence	Forward primer: cgc*ggatcc*atgagcttaacctctattaaac (BamH I)
	Reverse primer: gatagtacctacaatagccattaagattacctcctttgctt
PSMα4 sequence	Forward primer: aagcaaaggaggtaatcttaatggctattgtaggtactatc
	Reverse primer: ccg*gaattc*ttattttgcgaaaatgtcg (EcoR I)
