# Supplementary material for: Phenol-soluble modulin α4 mediates Staphylococcus aureus-associated vascular leakage by stimulating heparin-binding protein release from neutrophils
Source: Sci Rep. 2016 Jul 7;6:29373. doi: 10.1038/srep29373 (PMC4935938; doi:10.1038/srep29373)
Supplement: Supplementary Information [file srep29373-s1.pdf]

Phenol-soluble modulin α4 mediates *Staphylococcus aureus*-associated vascular leakage by stimulating heparin-binding protein release from neutrophils

Lin li[1], Yaya Pian[3], Shaolong Chen[1], Huaijie Hao[4], Yuling Zheng[1], Li Zhu[5], Bin Xu[6], Keke Liu[1], Min Li[2#], Hua Jiang[1#]&Yongqiang Jiang[1#]

[1]State Key Laboratory of Pathogen and Biosecurity, Institute of Microbiology and Epidemiology, Academy of Military Medical Sciences, Beijing, China

[2]Department of laboratory medicine，Renji Hospital，School of Medicine，Shanghai Jiao Tong University, Shanghai, China.

[3]Key Laboratory of infection and immunity, Institute of Biophysics, Chinese Academy of Science, Beijing100101, China

[4]Institution of Microbiology, Chinese Academy of Sciences, Beijing, China

[5]State Key Laboratory of Pathogen and Biosecurity, Beijing Institute of Biotechnology，Beijing100071, China

[6]National Center of Biomedical Analysis, Beijing, China,

# To whom correspondence should be addressed.

Correspondence and requests for materials should be addressed to M.L.(ruth_limin@126.com) or H.J. (email: jhua76@126.com) or Y.Q.J. (email: jiangyq@bmi.ac.cn).

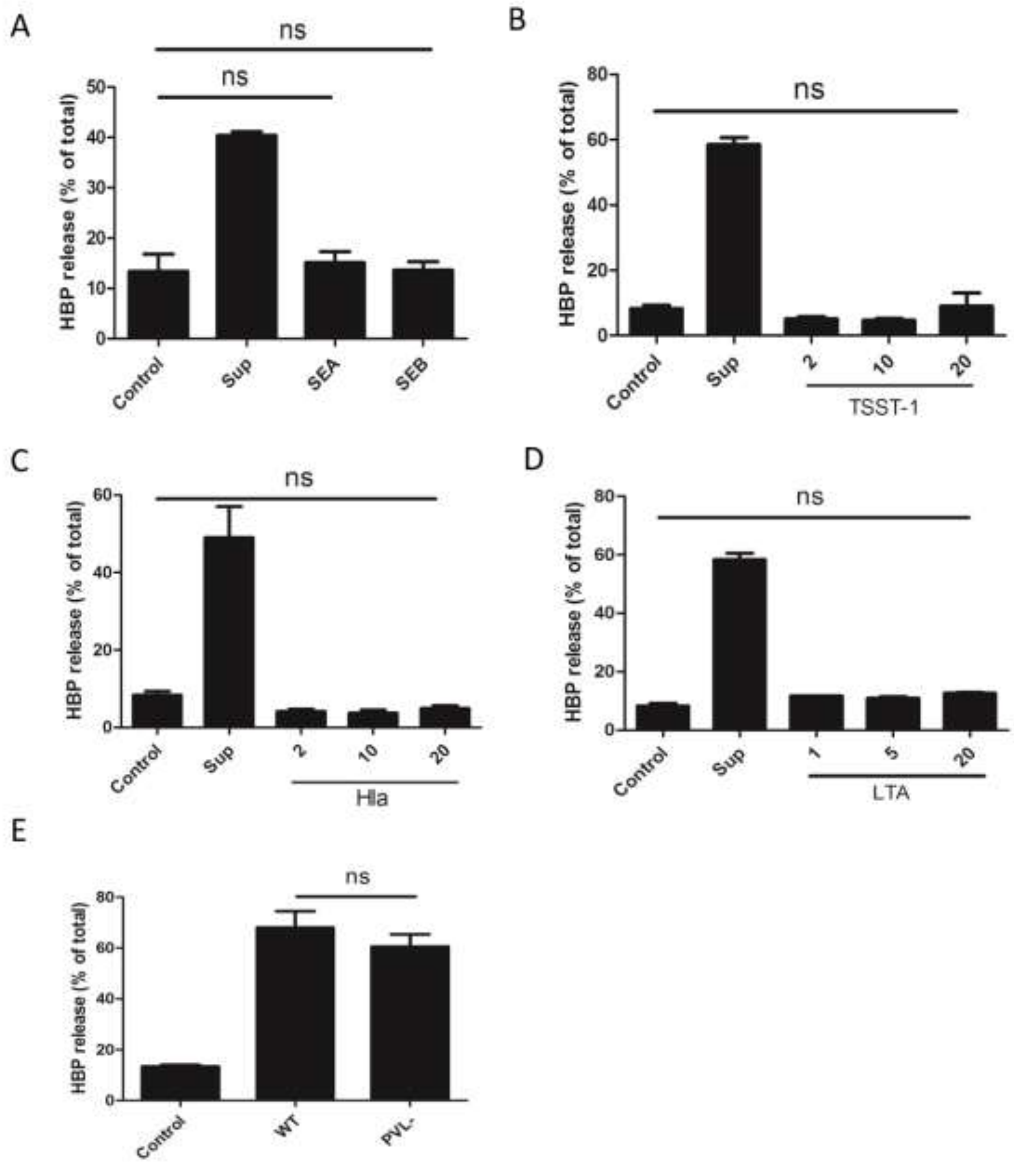

Supplementary Figure 1: Well recognized virulence factors did not induce HBP release.

(a) Superantigen staphylococcal enterotoxin (SE) A and SEB (20μg/mL); (b) Toxic shock syndrome toxin (TSST-1, 2, 10, 20μg/mL); (c) Hemolysin-α (Hla, 2, 10, 20μg/mL); (d) cell wall composition, lipoteichoic acid (LTA, 1, 5, 20μg/mL) did not induce HBP

release from whole blood of healthy donors. Human whole blood (10% in the final volume of 1ml) was incubated with the each virulence factor, respectively, at 37ºC for 30 minutes. One hundred µL supernatant was collected and HBP levels in the supernatant were detected by ELISA. (e) The supernatants of PVL-knockout mutant and the WT strains showed no significant difference in HBP release.

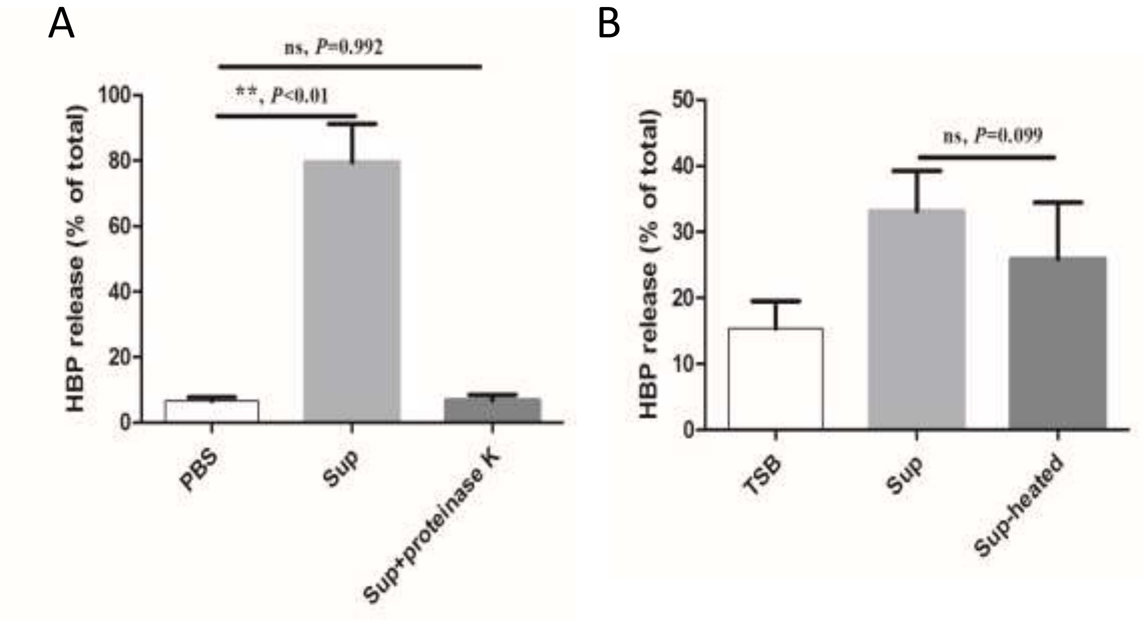

Supplementary Figure 2. USA300 supernatant induces HBP release from whole blood in a proteinase K-sensitive and heat-insensitive manner

A. Proteinase K treatment completely abolished supernatant-induced HBP release from whole blood. The supernatant was treated with 100 µg/mL proteinase K at 37°C for 1 h. **B.** Heat treatment of the supernatant (100°C for 15 min) did not significantly affect the induction of HBP release.

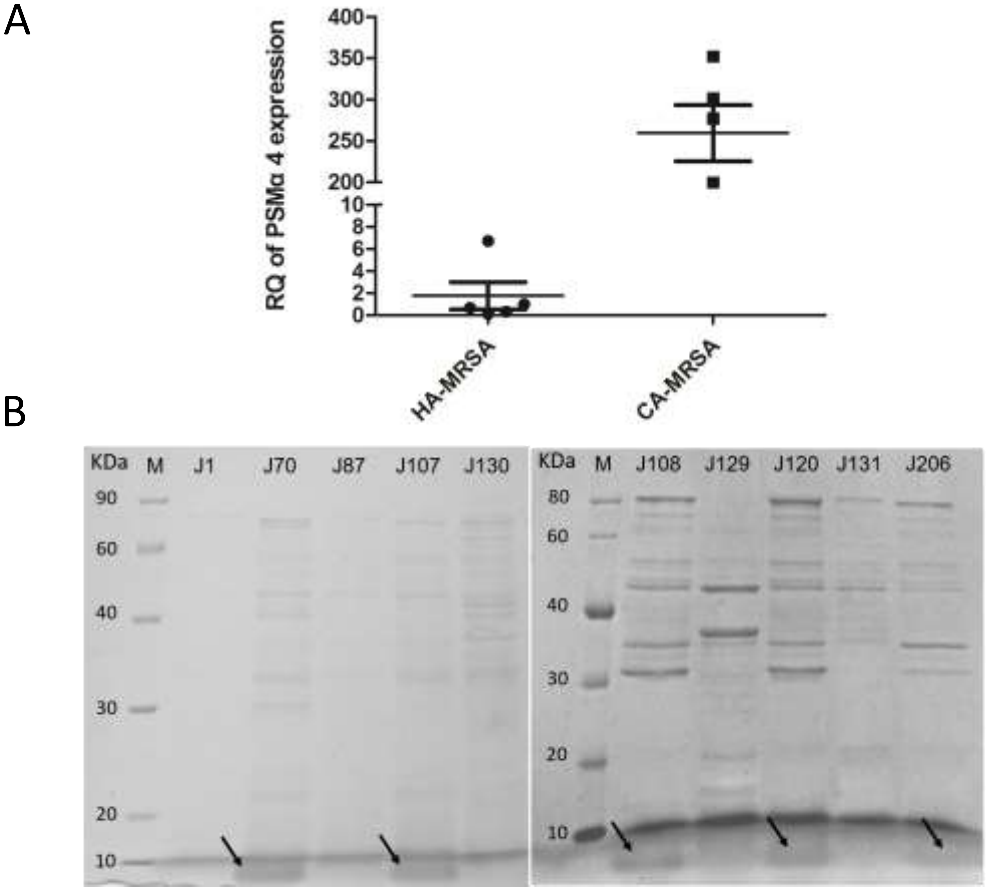

Supplementary Figure 3. The expression of PSMα in CA-MRSA and HA-MRSA was evaluated.

Five HA-MRSA strains (J1, J87, J129, J130, J131) and five CA-MRSA strains (J70, J107, J108, J120, J206) were chosen to evaluated the PSMα4 and PSM**α** expression. Transcription level of PSM**α4** was evaluated by qRT-PCR (a) and Translation level of PSM**α** was evaluated by 12% SDS-PAGE (b).

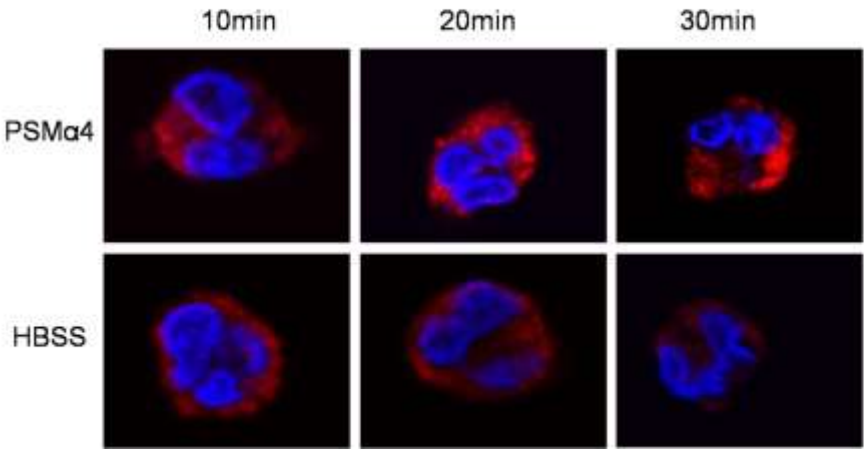

Supplementary Figure 4. PSMα4 induces CD63 translocation from the cytoplasm to the cell membrane

Human PMNs were incubated with 10 μg/mL PSMα4 at 37ºC for 10, 20, or 30 min, stained with mouse monoclonal antibody against CD63 and TRICT-conjugated anti CD63 antibody (red) and DAPI (blue), and analyzed by confocal microscopy.

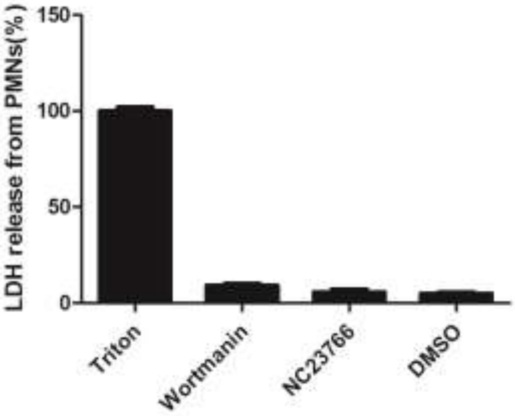

Supplementary Figure 5. The PI3K and Rac inhibitors show no cytotoxicity on PMNs. PMNs were incubated with 1 μM wortmannin, 50 μM NSC23766, or equal amounts of DMSO at 37°C for 1 h. The supernatant was collected and LDH release was measured. ***P < 0.001, **P < 0.01, *P < 0.05.

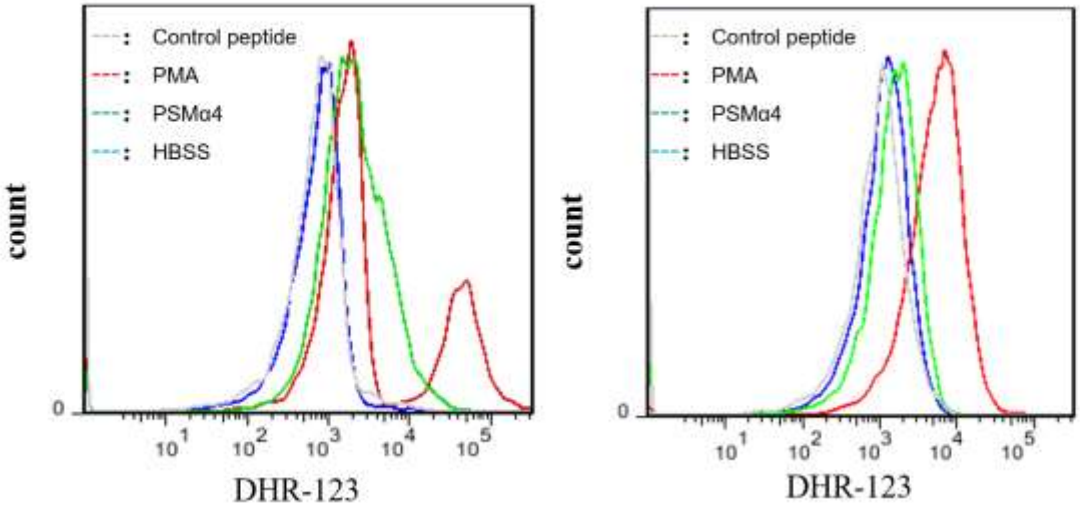

Supplementary Figure 6. PSMα4 stimulates ROS production from PMNs. PMNs were incubated with 10 μg/mL of PSMα4 without (A) or with 10% serum (B). ROS production was detected by flow cytometry, and PMA was used as the positive control.
